# Invasion History of *Sirex noctilio* Based on *COI* Sequence: The First Six Years in China

**DOI:** 10.3390/insects11020111

**Published:** 2020-02-09

**Authors:** Xueting Sun, Jing Tao, Alain Roques, Youqing Luo

**Affiliations:** 1 Sino-France Joint Laboratory for Invasive Forest Pests in Eurasia, Beijing Forestry University, Beijing 100083, China; xuetingsun507@126.com (X.S.); taojing1029@hotmail.com (J.T.); 2 Sino-France Joint Laboratory for Invasive Forest Pests in Eurasia, INRAE, UR0633 Orléans, France

**Keywords:** *Sirex noctilio*, invasive species, genetic structure, haplotype network, mitochondrial marker

## Abstract

*Sirex noctilio* F. (Hymenoptera: Siricidae: Siricinae), a new invasive species in China, is a significant international forestry pest which, transported via logs and related wood packing materials, has led to environmental damage and substantial economic loss in many countries around the world. It was first detected in China in 2013, and since then infestations have been found in 18 additional sites. Using a 322 bp fragment of the mitochondrial barcode gene *COI*, we studied the genetic diversity and structure of *S. noctilio* populations in both native and invaded ranges, with a specific focus in China. Twelve haplotypes were found across the native and invaded distribution of the pest, of which three were dominant; among these there were only one or two mutational steps between each pair of haplotypes. No obvious genetic structure was found other than in Chinese populations. China has a unique and dominant haplotype not found elsewhere, and compared with the rest of the world, the genetic structure of Chinese populations suggested a multiple invasion scenario.

## 1. Introduction

Biological invasions are increasing exponentially as globalization progresses, without any sign of saturation, especially in insects [1,2]. At present, phytophagous species associated with woody plants constitute the majority of these insect invaders [3]. The problem warrants increased attention, as the invasion of alien species is likely to impact native biodiversity, and can also cause huge economic losses [4,5,6,7]. There is a statistically significant correlation between the number of major invasive alien species in China and Chinese trade imports from the year 1980 to 2015 (Pearson correlation r = 0.915, *p* < 0.01, N = 35). The current primary route for alien forest species invading China is through human activity. This is mainly unintentional introduction; transported seedlings and their propagation materials carry 46% of human activity-generated invaders, with packaging materials or wood accounting for 21% (National Bureau of Statistics, http://www.stats.gov.cn/tjsj/tjgb/ndtjgb/) [8]. Some major invasive species, such as the North American bark beetle *Dendroctonus valens*, fall webworm *Hyphantria cunea* and loblolly pine mealybug *Oracella acuta*, have already caused the destruction of forestry resources in China, negatively influencing species diversity and ecological security, and resulting in major economic loss [9,10,11]. In order to monitor and manage the invasion of a pest, including possible biocontrol by natural enemies as well as the prevention of further introductions, it is essential to figure out its invasion history. However, observational records are usually difficult to get, and often biased, particularly when considering a recently-arrived invasive species. From this perspective, genetic analyses comparing populations in the native range with those in the invaded areas constitute an important tool for reconstructing the invasion history.

Molecular markers, such as mitochondrial, ribosomal, chloroplast, and microsatellite DNA, and others, have become increasingly popular for investigating phylogenetic relationships between insect populations. By using mitochondrial genes and microsatellite loci, Wei (2013) showed that the invasive diamondback moth, *Plutella xylostella*, moves within China from south to north, with rare effective travel in the reverse direction [12]. Similar patterns are found for the oriental fruit moth, *Grapholita molesta*, which originates in southern China and has dispersed to the north [13]. In the case of the western flower thrips, *Frankliniella occidentalis*, Cai (2017) recently determined the 13-year history of its invasion of China, revealing multiple introductions and further human-assisted spread, using mitochondrial genes and 18 microsatellite loci. In order to infer the invasion routes of the Asian long-horned beetle (*Anoplophora glabripennis*) in Europe, Javal (2019) used a 485 bp fragment of the mitochondrial barcode gene *COI*, which revealed multiple introductions from China and a bridgehead effect [14]. The *COI* gene (mtDNA cytochrome oxidase I) has been of widespread use in tracing invasions [15,16,17,18].

*Sirex noctilio*, a woodwasp native to Eurasia and North Africa, is a significant forestry pest, subject to quarantine regulations around the world [19]. Its larvae tunnel the trunks of conifer trees and infect the wood with a damaging fungus, ultimately resulting in tree death. The first record of *S. noctilio* outside its native Eurasian range was in the southern hemisphere during the 1900s [20]. Following introduction to New Zealand, it spread rapidly through the stands of *Pinus radiata* planted there, killing trees across approximately 120,000 ha of the north–central region and thereby causing huge economic and ecological loss. During 1987–1989, an outbreak in Australia and Tasmania killed more than 5 million *P. radiata* trees [21]; it was first found there 35 years ago [22]. Several areas of South America have also been attacked, including Uruguay in 1980, Argentina in 1985, Brazil in 1988, and Chile in 2000. The mortality rate of pine trees was high, at 60% and 80% in Argentina and Uruguay, respectively. In Brazil, a 350, 000 ha pine plantation was destroyed. In 1994, it was reported in South Africa and in 2006 in the USA and Canada.

In China, *S. noctilio* was first detected in 2013 in the northeast, at Daqing, Heilongjiang Province [23,24]. Six subsequent years of field investigations uncovered 18 new occurrences, but these were followed by a post-invasion latency period, or time lag. Therefore, the first place where alien forest pests are found does not necessarily represent their point of invasion. However, the relationship between the initial discovery site and the invasion site is typically the closest and most direct. It is generally the case that the site of initial discovery contains the invading colony [8]. Moreover, when the invasion site’s latitude and habitat are similar to those of the pest’s origin, the possibility of invasion and colonization is increased [8]. *S. noctilio* is found in China at similar latitudes to its native sites; moreover, its native regions in Russia are near the Chinese border. Based on climatic conditions and host distribution, domestic and foreign experts analyzed the potential woodwasp distribution area in China. Their results indicated that a band stretching from Yunnan to Heilongjiang represents a highly suitable area for its establishment [25]. Its potential host tree species are widely distributed in China; hence, there is a high potential invasion risk into additional areas.

In order to investigate the origins and pathways of invasion, Bernard (2012) used microsatellite and sequence data collected from five continents. Two major sources were identified, although the invasion history was quite complex, with most of the populations containing admixtures of independently introduced European insects that subsequently spread through the invaded regions. Similarly, Bittner (2017) investigated the genetic diversity of 924 *S. noctilio* in nine populations from New York and Pennsylvania (US), Ontario (Canada), and Queensland (Australia), showing that multiple introductions occurred in northeastern North America. However, a more comprehensive understanding requires the collection of more samples from its native Europe and Asia.

In this study, the genetic structure and invasion routes of *S. noctilio* populations were surveyed using the mtDNA cytochrome oxidase 1 (*COI*) gene. Our objective was to investigate the genetic structure and genetic diversity of *S. noctilio* populations around the world, especially in relation to Chinese infestations. We compared patterns in invaded continents and native regions, analyzing for the first time 778 individuals from 6 continents. In particular, samples from original European populations and recent Chinese infestations were included. Overall, this study aims to elucidate the invasive routes of this pest as well as provide data enabling the prevention of the harmful consequences of *S. noctilio* spread.

## 2. Materials and Methods

### 2.1. Sample Collection and Selection

**Figure 1:**
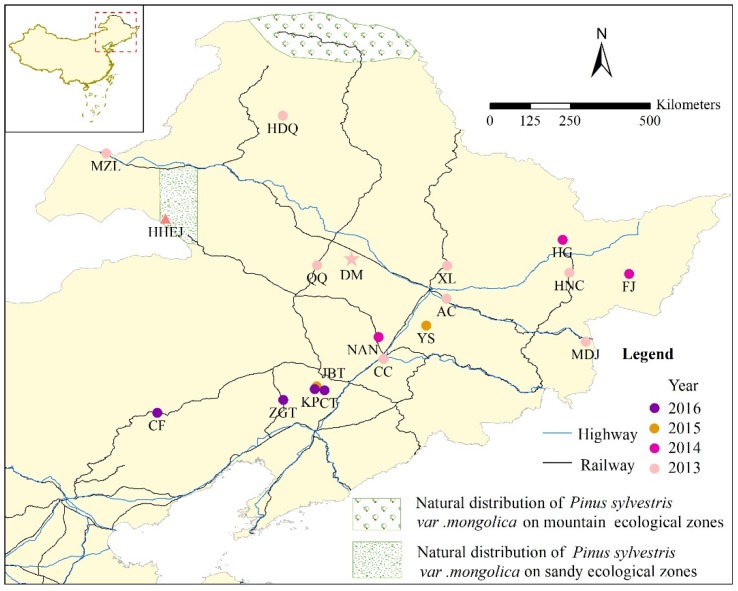
Sampled Chinese *S. noctilio* population locations (indicated by 2–4 letter combinations) and year of the first detection; natural distribution area of the host. The triangular marker for HHEJ indicates the Honghuaerji Scenic Area. The pentagram marker for DM indicates the place where *S. noctilio* was first detected in China.

*S. noctilio* samples were collected from native European, non-Chinese invaders, and Chinese populations, comprising 44, 127, and 607 specimens, respectively. A summary of sampling sites and common parameters of the genetic diversity of *S. noctilio* in this study is provided in Table 1. GenBank/DRYAD provided 23 of the 44 native European specimens; others were dry or anhydrous ethanol-preserved individuals borrowed from museums or donated by local institutions. Non-Chinese invader populations were represented by 22, 39, 50 and 16 GenBank/DRYAD samples from Africa, North America, South America, and Oceania, respectively. All Genbank/DRYAD samples are retrieved from DRYAD: https://datadryad.org/stash/dataset/doi:10.5061/dryad.37mm8; from Genebank: https://www.ncbi.nlm.nih.gov/nuccore/?term=sirex+noctilio. Finally, we obtained a total of 607 specimens from 19 Chinese populations (Table 1 and Figure 1), donated by various local forestry bureaus or obtained from the standard quarantine facility of Beijing Forestry University, which contains a large number of locally collected infested stems; these specimens were identified using taxonomic literature on *Sirex* woodwasps before DNA extraction [26]. As the Chinese source areas were infested at different times and to different degrees, the number of specimens and sampling year vary. Dried museum samples were handled outside our laboratory, as described below; specimens already in 99.7% pure anhydrous alcohol remained so; all remaining specimens were frozen at −80 °C.

As there is an obvious sample size bias towards the six Chinese populations with high sample numbers (population codes DM, QQ, HG, FJ, JBT, CT), we used only 20 of the sequenced individuals, which included all the haplotypes found in each population. We selected individuals from each haplotype based on the selection ratio and the number of samples in each haplotype. The final total of 364 samples for analysis consisted of 44, 127, and 193 individuals from Europe, non-Chinese invaded areas, and China, respectively.

### 2.2. DNA Sequence Analysis

For the native European samples not supplied as sequences directly from GenBank, DNA extraction, PCR amplification and sequencing of the barcode fragment were performed for 15 specimens at the Laboratory of Forest Zoology URZF, INRAE (Orléans, Paris, France). DNA extracts were prepared from one adult hind leg or thorax muscle using NucleoSpin tissue XS kits (Macherey Nagel, Dylan, Germany) according to the manufacturer’s protocol. The *COI* barcoding fragment was amplified via PCR using the forward primer LCO1490 (5ʹ-GGT CAA CAA ATC ATA AAG ATA TTG G-3’) and the reverse primer HC02198, (5’-TAA ACT TCA GGG TGA CCA AAA AAT CA-3’) (Vrijenhoek, Rijswijk, Netherlands, 1994). PCR reagents were used and reactions performed following Sun [24]. PCR products were purified using the QIAquick PCR Purification Kit (Qiagen, Hilden, Germany). Sequencing was performed using the Sanger method with an ABI Prism BigDye Terminator v3.1 cycle sequencing kit (Thermo-Fisher, Waltham, MA, USA). The reagents and procedure used were: 1 μL 5 × bigdye buffer, 1 μL bigdye, 1 μL LCO/HCO, diluted to 3.3 μM, 2 μL DNA; 30 cycles of 10 s at 96 °C, 5 s at 50 °C, 3 min at 60 °C. The remaining 6 specimens were borrowed from museums and had to remain intact; for these the same *COI* fragment was amplified at the Canadian Centre for DNA Barcoding (CCDB–Biodiversity Institute of Ontario, University of Guelph) using a slightly different sequencing set (C-LepFolF/C-LepFolR), following the standard high-throughput protocol [27].

DNA was extracted from the leg or thorax muscle of each Chinese specimen using the Multisource Genomic DNA Miniprep Kit (Axygen, San Francisco, CA, USA), following the manufacturer’s protocol to prevent cross-contamination, all tools were sterilized by flame or 75% pure anhydrous ethanol. DNA was eluted using 70 μL of elution buffer and stored at −20 °C until use. Species-specific *COI* amplification primers were synthesized by a commercial company (SinoGenoMax, Beijing, China). PCR primers LCO1490 and HC02198 were used as above. Reactions were performed in a total volume of 25 μL, containing 12.5 μL of 2 × GoTaq^®^ Green Master Mix (Promega, Madison, WI, USA), 1 μL of each primer, 1.5 μL of DNA template, and 9 μL of ultrapure water. The reaction procedure was as follows: initial denaturation for 2 min at 94 °C, followed by 35 cycles of denaturation at 94 °C for 30 s, annealing at 45 °C for 30 s, and extension at 68 °C for 2 min, with a final extension at 68 °C for 10 min. PCR products (3 μL) were analyzed by electrophoresis on a 1.5% (*w/v*) agarose gel (1 × TAE), alongside a DNA marker (D2000, Takara, Kyoto, Japan). After electrophoresis at 130 V for 20 min, the PCR products were visualized using ethidium bromide and ultraviolet light. Finally, products clearly visible after electrophoresis were sent to a commercial company (SinoGenoMax, China) for sequencing in both directions.

All electropherograms were checked manually in CodonCode Aligner v3.7.1. (CodonCode Corporation, Centerville, MA, USA) to assess their quality. The alignment was done independently using Clustal W implemented in MEGA v6.06 with default parameters, and no pseudogenes or stop codons were detected in the sequences [28,29]. All *COI* sequences were trimmed to the same length (322 bp) for final alignment.

Details on the collecting data for each specimen, as well as photographs, sequence records, trace files, and primer sequences used for PCR amplification, together with GenBank accession numbers, are available through the dataset in BOLD (www.boldsystems.org).

### 2.3. Statistical Analysis

Common parameters of genetic diversity, including the number of haplotypes, haplotype, and nucleotide diversity were calculated using DNAsp v6 [30]. The haplotype network was built in PopART [31,32,33]. Haplotype distribution and frequency were projected on a map using ArcGIS 9.3 (ESRI, Redlands, CA, USA).

Analyses of molecular variance (AMOVA) were performed on native European populations and Chinese populations to measure the partitioning of genetic variation between populations and groups of populations in Arlequin 3.5.2.2 [34,35]. The first AMOVA was performed on four clusters of Chinese populations in the northeast of China defined by geographic proximity: western (mountain area), middle (city area), which was divided into two parts, and eastern (city and mountain areas). A second AMOVA was performed on two clusters of Chinese populations grouped by latitude, north and south, in order to test the trend of spread.

## 3. Results

**Figure 2:**
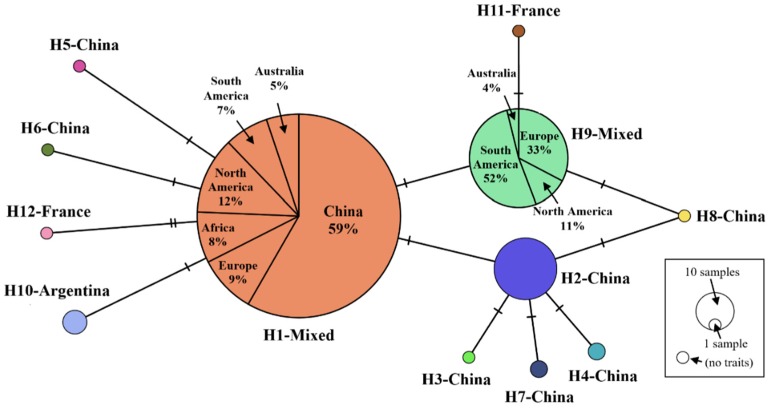
*S. noctilio* haplotype network based on 322 bp sequences, 364 samples around the world. Short solid line(s) between haplotypes show(s) the number of mutation sites.

**Figure 3:**
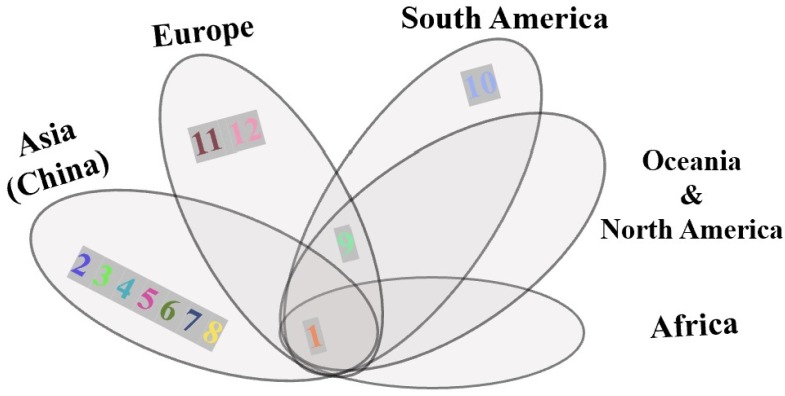
Venn diagram of *S. noctilio* haplotypes by continent. Numbers and colors correspond to the haplotype network shown in Figure 2.

**Figure 4:**
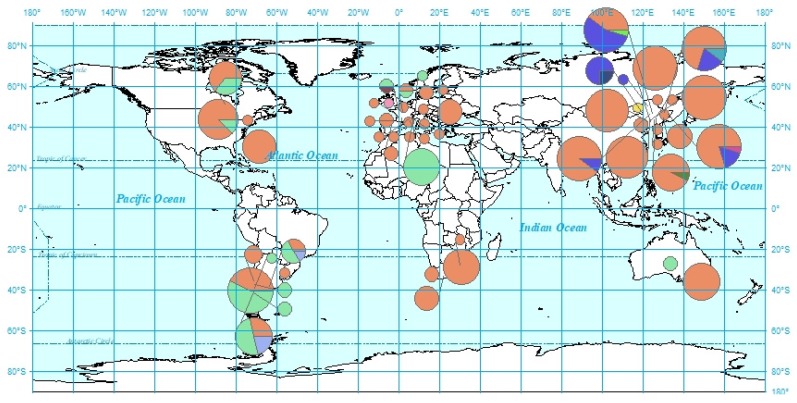
Global *S. noctilio* haplotype distribution. Colors correspond to the haplotype network shown in Figure 2.

There were 11 variable sites among all insects sampled. Three dominant haplotypes, H1, H2, and H9, were present among the total of 12 haplotypes; the haplotype diversity was 0.4207. Only one or two mutation sites existed across the different haplotypes (Figure 2). There were two mutation sites between H1 and H12. H1 and H9 were haplotypes that were made up of populations from different countries: H1 was widely located on all continents, whereas H9 was not found in China and Africa (Figure 2 and Figure 3). H2, a major Chinese haplotype along with H1, was found only in Chinese samples (Figure 2). H3, H4, H5, H6, H7, and H8 were unique to Chinese populations. H8, the haplotype found near the national border with Russia (Figure 4) is worth noting. H10 was unique to South America, as were H11 and H12 to Europe.

**Figure 5:**
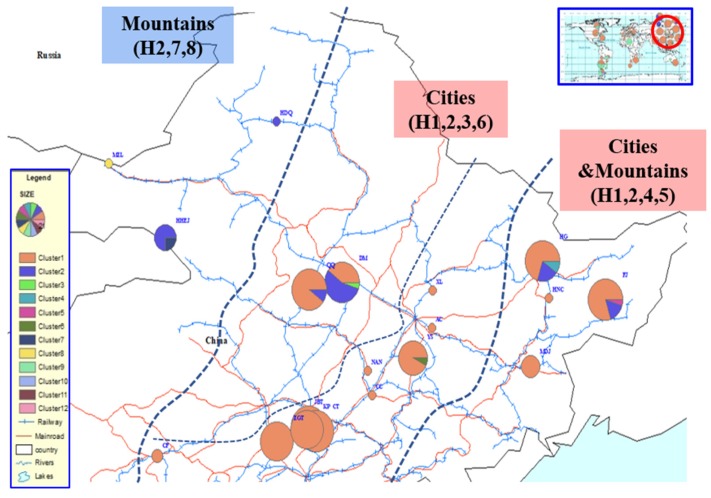
Chinese *S. noctilio* populations grouped by geographic proximity (west to east) and their haplotype projections.

**Figure 6:**
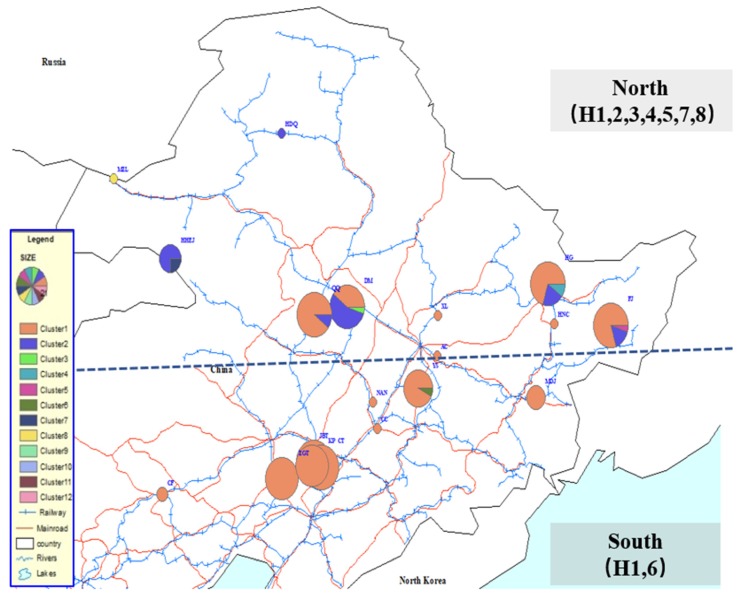
Chinese *S. noctilio* populations grouped by latitude (north to south) and their haplotype projections.

AMOVA analysis of the genetic structure of Chinese populations (Table 2) showed that when grouped by geographic proximity (Figure 5), most of the variation (38.64%, *p* < 0.01) was between geographic clusters, with a relatively small part of the genetic variance (9.15%, *p* < 0.01) between populations within geographic clusters. When sites were grouped by latitude (Figure 6), the variation (25.58%, *p* < 0.01) between clusters was slightly higher than that (22.46%, *p* < 0.01) between populations within clusters. Within this study, no clear genetic structure emerged within native European populations.

The Chinese sampling sites are divided into four groups based on geographical proximity: west for one group, middle for two groups and east for one group. The middle and eastern groups, where there are cities, contained 4 haplotypes, whereas the western group, which is mainly mountainous, had 3 haplotypes. The first site discovered in China, DM, contained 40% H1 and a similar proportion, 55%, of H2. There is no H1 in the western group, although the proportion of H1 in the two middle area groups is quite high as is also found in the eastern group. There is a decreasing trend from west to east in the proportion of H2. The representative populations on this axis were HDQ, DM, and HG with 100%, 55%, and 20% H2, respectively. Among the other six unique haplotypes in China, H8 is located in MZL, on the border between China and Russia. Four of the remaining five haplotypes are distributed in the populations from which large samples were taken and are located in city areas. The sixth, HHEJ, is located in the area from which *P. sylvestris* var. *mongolica* seedlings are taken for distribution and planting elsewhere. H3 is present in 5% of DM individuals, and so does H4, H5, H6, H7, these haplotypes occurred in 10%, 5%, 7%, and 25% of sampled insects of HG, FJ, YS and HHEJ, respectively. The latitude 46.204°N was used to group samples into the categories north and south. Haplotype diversity in the northern area is obviously higher than that in the south. The northern cluster has seven haplotypes, H1, H2, H3, H4, H5, H7, and H8, from nine populations, compared to two haplotypes (H1 and H6) from ten populations in southern China.

## 4. Discussion

### 4.1. Host Unity of S. Noctilio in China

The host range of *S. noctilio* is rather large, although it is known that the pest is harmful to various kinds of conifers around the world, especially *Pinus*, *Picea*, *Abies*, *Larix*, and *Pseudotsuga* [19]. Both natural forests and pine plantations can suffer damage, especially when they are overstocked and stressed. In China, the origin of *P. sylvestris* var. *mongolica* is in the area of Honghuaerji in Inner Mongolia and north of the Huma River in Heilongjiang [36]. Field investigation found no *S. noctilio* in the wild distribution areas of *P. sylvestris* var. *mongolica* in either mountain or sandy ecological zones (Figure 1), other than two larvae found in the western, sandy area. This occurrence, and the unique haplotype, probably came from the considerable imports of Russian timber required for the construction of Honghuaerji Scenic Area.

Currently, the main infestation zones in China are those where *P. sylvestris* var. *mongolica* has been introduced. Two typical examples are Hegang (Heilongjiang) and Jinbaotun (Inner Mongolia). The former is a mixed forest of *Pinus koraiensis*, *L. olgensis*, *Picea koraiensis* and *P. sylvestris* var. *mongolica*. The latter contains *Pinus tabuliformis* and *P. sylvestris* var. *mongolica*. However, fieldwork has indicated that the only infested conifer species in China to date is *P. sylvestris* var. *mongolica*, even when other potential hosts are present in the same forest [37]. As is characteristic of invasive pests, *S. noctilio* appears to be harmless in the natural distribution area of its favorite host tree species.

### 4.2. Patterns of Multiple Invasion and Spread in China

There are currently three more frequent haplotypes, H1, H2, and H9, which have a worldwide distribution. It is thus unsurprising that they are in the center of the haplotype network and closely connected with other well-established haplotypes [38]. Interestingly, H1, despite being globally the most prevalent haplotype, is absent in western China, although strongly represented in the city regions of central and eastern China. This constitutes evidence that genetic communication is human activity-derived in both invasion and spread scenarios. Given that the western component is the primary seedling source of *P. sylvestris* var. *mongolica*, trees from this area will be planted elsewhere. We speculate that H1 is an invasion haplotype carried by humans that have entered the central and eastern urban areas of China. As it existed on all continents, its source is unclear. Based on our data, H2, H7 and H8 were found only in China, and given that they are unlikely to have originated there, we are unable to identify their origin. H2 is the main haplotype of the western component, with a distribution ratio that gradually decreases from west to east; this is in line with the characteristics of spread from a point of origin to other regions. This, as noted above, may represent an invasion originating with Russian wood imported for construction at Honghuaerji and spread eastward by planting. Severely endangered woodlands in Dumeng, Hegang, Fujin, and other eastern areas may have been invaded in this way. H7 and H8 are also unique to the western region; H8 occurs on the border with Russia.

Grouped by latitude, significantly more haplotypes exist in northern Chinese *S. noctilio* populations than in those to the south. Damage to woodlands in the northeastern region occurs discretely, in multiple forest farms, parks, and other wooded land. This damage was concentrated, as is characteristic of the middle to late stages of an invasion. In contrast, in newly-invaded southern areas, such as Jinbaotun, damaged forests were localized and individual affected trees were distributed discretely in the affected area, indicating the initial stage of introduction. Although the 1130 km between Honghuaerji and Jinbaotun is shorter than the 1400 km between Honghuaerji and the affected area in the northeast, as the haplotype H1 found in the southern region is not present in Honghuaerji, the south regional populations may originate from northern China.

*S. noctilio* has a relatively strong natural flight ability. In the southern hemisphere, the maximum spread rate of an invading population of *S. noctilio* is 82 km per year [39]. In China, the diffusion rate of the pest is 74.57 km/year [40]. The straight-line distances between Dumeng, the first site detected, and the other two severely endangered areas, Hegang and Jinbaotun, are 440 km and 390 km, respectively, which would take about 6 years to achieve at 74.57 km/year. Yet the woodwasp was already present in 2014 and 2015. The first recorded occurrence sites in all the affected areas are distributed along with the main traffic networks (Figure 1), which thus appear to have increased the impact caused by human activities, such as wood packaging and log transportation.

### 4.3. Focus for Further Research

When analyzing biological invasions, knowledge of the genetic structure of populations from the area of origin is vital in determining the source. Our target species is native to Eurasia and North Africa, but it does not cause serious harm there and has consequently received less attention, leading to difficulties in collecting samples. In particular, the lack of samples from the Russian region caused sampling breakpoints between the European and northeast Chinese populations. The observation that H2 occurred only in China may be due to the large sample size from some previously unsampled sites from this area. More reliable results may be obtained from sampling efforts focused on this region and on western China. Finally, microsatellite marker use involving cytochrome C oxidase subunit I (*COI*) could prove effective, showing its utility as a method for determining invasion pathways and spread.

## 5. Conclusions

In this study, a 322 bp fragment of the mitochondrial barcode gene *COI* was used to analysis the genetic diversity and structure of *S. noctilio* populations in both native and invaded ranges, with a specific focus in China. Twelve haplotypes were found across the native and invaded distribution of the pest, of which three were dominant; among these there were only one or two mutational steps between each pair of haplotypes. No obvious genetic structure was found other than in Chinese populations. China has a unique and dominant haplotype not found elsewhere, and compared with the rest of the world, the genetic structure of Chinese populations suggested a multiple invasion scenario.

## Figures and Tables

**Table 1:** Summary of sampling sites and common parameters of genetic diversity of *Sirex noctilio* analyzed in this study.

Locality	Population Code	First Detection	Sampling Time	N(Sequenced Individuals)	N variable Sites	Nht	h	π
Total				364(778)				
Asia, China				193(607)	7	8	0.311	0.00114
Dumeng, Heilongjiang	**DM**	**2013**	**2016**	**20(66)**	**2**	**3**	**0.549**	**0.00183**
Qiqihaer, Heilongjiang	**QQ**	**2013**	**2016**	**20(64)**	**1**	**2**	**0.185**	**0.00057**
Hegang, Heilongjiang	**HG**	**2014**	**2015-16**	**20(204)**	**2**	**3**	**0.472**	**0.00191**
Fujin, Heilongjiang	**FJ**	**2014**	**2015**	**20(69)**	**2**	**3**	**0.344**	**0.00111**
Hulunbeier, Inner Mongolia	**HHEJ, HLBE**	**2014**	**2014**	**8**	**1**	**2**	**0.4**	**0.00124**
Keyihe, Inner Mongolia	**HDQ**	**2013**	**2013**	**1**	**0**	**1**	**0**	**0**
Yushu, Jilin	**YS**	**2015**	**2015**	**14**	**1**	**2**	**0.138**	**0.00043**
Jinbaotun, Inner Mongolia	**JBT**	**2015**	**2015-16**	**20(83)**	**0**	**1**	**0**	**0**
Changtu, Liaoning	**CT**	**2016**	**2016**	**20(48)**	**0**	**1**	**0**	**0**
Changchun, Jilin	**CC**	**2013**	**2013**	**1**	**0**	**1**	**0**	**0**
Zhanggutai, Liaoning	**ZGT**	**2016**	**2016**	**18**	**0**	**1**	**0**	**0**
Kangping, Liaoning	**KP**	**2016**	**2016**	**18**	**0**	**1**	**0**	**0**
Chifeng, Inner Mongolia	**CF**	**2016**	**2016**	**2**	**0**	**1**	**0**	**0**
Acheng, Heilongjiang	**AC**	**2013**	**2013**	**1**	**0**	**1**	**0**	**0**
Mudanjiang, Heilongjiang	**MDJ**	**2013**	**2013**	**6**	**0**	**1**	**0**	**0**
	Ningan, Heilongjiang MDJ-DJ		2013					
	Suiyang, Heilongjiang MDJ-SY		2013					
	Muleng, Heilongjiang MDJ-BMT		2013					
	Hailin, Heilongjiang MDJ-CH		2013					
	Mudanjiang, Heilongjiang MDJ-SGLK		2013					
	Mudanjiang, Heilongjiang MDJ-XS		2013					
Harbin, Heilongjiang	**XL**	**2013**	**2013**	**1**	**0**	**1**	**0**	**0**
Nongan, Jilin	**NAN**	**2014**	**2014**	**1**	**0**	**1**	**0**	**0**
Jiamusi, Heilongjiang	**HNC**	**2013**	**2013**	**1**	**0**	**1**	**0**	**0**
Manzhouli, Inner Mongolia	**MZL**	**2013**	**2013**	**1**	**0**	**1**	**0**	**0**
Africa				22	0	1	0	0
South Africa	**SA**			**1**	**0**	**1**	**0**	**0**
Natal Midlands	NM			13	0	1	0	0
Western Cape	WC			6	0	1	0	0
Eastern Cape	EC			2	0	1	0	0
North America				39	1	2	0.264	0.00082
Canada	Can			27	1	2	0.352	0.00109
Canada-DRYAD	Can							
USA	NY, USA			12	0	1	0	0
USA-DRYAD	USA				0	1	0	0
South America				50	2	3	0.563	0.00202
Argentina (unknow)	Arg			25	2	3	0.568	0.00237
Arg-DRYAD	Arg, ArgES							
Arg-Arroyo del medio	**Arg**		**2018**					
Arg-Aluminé	**Arg**		**2018**					
Arg-Andacollo	**Arg**		**2019**					
Uruguay	Uruguay			1	0	1	0	0
Chile	Chi, Chil			24	1	2	0.511	0.00159
Chil-DRYAD	Chi, ChiS							
Oceania				16	1	2	0.226	0.0007
Australia	Austra, Austr			16	1	2	0.226	0.0007
Austr-DRYAD	AustrES, AustrEB							
Europe				44	4	4	0.533	0.00194
Switzerland	Switz			15	0	1	0	0
Swit-DRYAD	Swit, SW, Swi							
*Italy*	*Ita-XS*			*1*	*0*	*1*	*0*	*0*
*Poland*	*Pol-XS*			*1*	*0*	*1*	*0*	*0*
Hungary	**Hun**, Hun-XS		**2018**	**4**,2	0	1	0	0
Ger-Berlin	Ger-Ber			2	0	1	0	0
Ger-Stuttgart	Ger-Stu			1	0	1	0	0
Spain	Spain			3	0	1	0	0
Spain-DRYAD	Spain							
*Fr-Labenne*	**Fr6-Lab.**, *Fr-Lab.XS30*		**2017**	**1**,*1*	0	1	0	0
Fr-Meolans Revel	**Fr03-MR**			**1**	**0**	**1**	**0**	**0**
Fr-Villes sur Auzon	**Fr01-V.S.A**			**1**	**0**	**1**	**0**	**0**
Fr-Marseille	**Fr3-Mar.**		**2013**	**1**	**0**	**1**	**0**	**0**
Fr-Tarascon	**Fr11-Tara**		**2017**	**1**	**0**	**1**	**0**	**0**
*Fr-Brain sur Allones*	**Fr8-B.S.A.**, *Fr-B.S.A.XS31*		**2017**	**1**,*1*	1	2	0.667	0.00207
Fr-Etaules	**Fr4-Eta.**		**2017**	**1**	**0**	**1**	**0**	**0**
Fr-Lavercantière	**Fr18-Lav.**		**2011**	**1**	**0**	**1**	**0**	**0**
Fr-Orléans	**Fr21-Orle, Fr87-Orle**		**2017.2**	**2**	**1**	**2**	**0.667**	**0.00207**
Fr-St Alban des Hurtiè	**Fr16-St.Al**		**2017**	**1**	**0**	**1**	**0**	**0**
Fr-Roissy CDG	**Fr33-CDG**		**2017**	**1**	**0**	**1**	**0**	**0**
Fr-Tartigny	**Fr12-Tart**		**2016**	**1**	**0**	**1**	**0**	**0**

Numbers in parentheses are the number of actual sampled individuals from Chinese populations. Others are the sequences of the mitochondrial Cytochrome Oxydase subunit I (*COI*) gene used in this study based on 322 bp fragment. In bold, the specimens are amplified in our Chinese and French laboratories; Italics are the specimens amplified at the Canadian Centre for DNA Barcoding; others are retrieved from databases. n ht Number of haplotypes, h Haplotype diversity, π Nucleotide diversity.

**Table 2:** AMOVA analysis of genetic variation.

Source of Variation	d.f.	Sum of Squares	Variance Components	Percentage of Variation	*P*
*AMOVA—Chinese populations grouped by geographic proximity (West-East)*					
Among groups	3	11.561	0.08286	38.64 ***	0.006
Among populations within groups	15	4.256	0.01961	9.15 ***	0.003
Within populations	174	19.479	0.11195	52.21 ***	0.000
*AMOVA—Chinese populations grouped by latitude (North-South)*					
Among groups	1	6.262	0.05510	25.58 ***	0.004
Among populations within groups	17	9.555	0.04838	22.46 ***	0.000
Within populations	174	19.479	0.11195	51.97 ***	0.000

*** *p* < 0.01; ** *p* < 0.05; * *p* < 0.1.
